# Food Cue Reactivity and the Brain-Heart Axis During Cognitive Stress Following Clinically Relevant Weight Loss

**DOI:** 10.3389/fnut.2018.00135

**Published:** 2019-01-04

**Authors:** Henri G. Laurie Rauch, David J. Hume, Fleur M. Howells, Jacolene Kroff, Estelle Victoria Lambert

**Affiliations:** ^1^Division of Exercise Science and Sports Medicine, Department of Human Biology, Faculty of Health Sciences, University of Cape Town, Cape Town, South Africa; ^2^Department of Psychiatry and Mental Health, Faculty of Health Sciences, University of Cape Town, Cape Town, South Africa

**Keywords:** desire to eat, attention, ERP, Stroop, stress-induced eating

## Abstract

Successful weight loss maintainers are more vulnerable to stress induced eating. The aim of our study was to determine what effect an attention-demanding cognitive performance task had on brain-heart reactivity to visual food cues in women who maintained clinically relevant weight loss vs. women who had never weight cycled. A clinical weight loss group (CWL, *n* = 17) and a BMI-matched control group (CTL, *n* = 23) completed modified Stroop tasks that either included high calorie food pictures (Food Stroop) or excluded food cues (Office Stroop). ECG, breathing rate, and EEG were recorded. *CWL participants*: The Eating Restraint scores (Three Factor Eating Questionnaire) of the CWL participants correlated negatively with their heart rates recorded during the Food Stroop task (*r* = 0.62, *p* < 0.01). There was no such relationship in CTL participants. The P200 latencies in CWL participants evoked by the Stroop color-word cues at the C3 electrode were positively correlated to the log high frequency power in their cardiac spectrograms during the Food Stroop (*r* = 0.63, *p* < 0.02). There were no such relationships in the Office Stroop task nor in CTL participants. *Combined Group*s: Participants' heart rates were significantly lower (*p* < 0.05) and their RMSSD values and the log Total Power in their cardiac spectrograms were significantly greater during the Food Stroop vs. Office Stroop (*p* < 0.01, Bonferroni corrected). In conclusion Eating Restraint scores in CWL participants correlated with their Stroop heart rates, while the P200 latencies evoked by the Stroop cues correlated with the log high frequency power in their cardiac spectrograms (marker of cardiac vagal activation) during the Food Stroop task. This provides evidence that even 12 months after successful weight loss maintenance the cardiac ANS reactivity to food cues while completing a cognitive performance test was still different to that in individuals of normal weight who never weight cycled. Across all participants the cardiac ANS reactivity evoked by performing the Stroop task was lowered by food cues suggesting that the dampening effect of food cues on cardiac ANS reactivity may be one of the drivers of ‘stress induced' eating.

## Introduction

The increasing rates of obesity worldwide has been attributed to the increased abundance of processed foods at more affordable pricing (1), further stimulated by effective marketing campaigns (2) and an overabundance of environmental food cues (3) found to be as enticing as real food exposure (4). In this regard real food exposure has been found to be associated with anticipatory autonomic nervous system (ANS) responses like increased heart rate, blood pressure (BP) and skin response (5–7) and decreased heart rate variability (HRV) (8). These anticipatory ANS responses are needed to prepare the body for foraging behavior needed to obtain food (9). The rationale for the current study was to examine the effect of cognitive stress on the anticipatory ANS responses to food cue exposure. We used the Stroop task as a cognitive stressor, because Stroop color-word conflict task performance has previously been found to be associated with increased heart rates, BP and respiration rates in participants (10).

These anticipatory increases in heart rate and BP observed during food cue exposure and during completion of the Stroop task, is thought to be modulated by the “central autonomic network” (CAN). Given the impact that this network, that incorporates the insular cortex, anterior cingulate cortex and amygdala (11, 12), has on heart rate, it has been aptly called the brain-heart axis (11). The CAN increases heart rates via dorsomedial hypothalamus activation of sympathetic nerves that also effects increases in blood pressure and respiration rates (13, 14).

Two further drivers of obesity in our modern fast paced lifestyles are low physical activity levels (15) and high stress levels (16–18). Stress, in turn, has been associated with ingestion of highly processed foods to improve mood and to provide psychological comfort (19, 20). The link between peripheral physiology (i.e., food processing in the gut) and improved mood/psychological comfort is provided by brain-gut modulation via the rostral forebrain (21, 22). These associations between food and mood/comfort links food cues and eating behavior (23).

Our highly obesogenic environments then necessitates more extreme weight control strategies in successful weight loss maintainers compared to normal weight individuals (24). Indeed successful long-term weight loss maintainers have been found to influence their eating behavior by engaging in frequent self-monitoring of body weight and food intake (25), however, this may come at the price of high levels of eating restraint (26). In this regard, female restrained eaters report increased baseline cortisol levels (27), which suggests of a greater vulnerability to stress induced eating. However, rather than this being a simple one-to-one relationship (28), restrained eaters have been found to succumb when allocating cognitive resources to an attention-demanding task that overstretch their ability to monitor dietary restraint (29).

In addition, the sensory brain areas in successful weight loss maintainers have been found to be more reactive to food cues as compared to normal weight and obese individuals (30). This suggests upregulated anticipatory reward processes in weight loss maintainers that leads to greater inhibitory processing (i.e., eating restraint) to prevent overindulgence (30). One such sensory brain area, found to have significantly elevated reactivity to food cues in successful weight loss maintainers, is the insula cortex. The insula cortex is not only a key sensory area that monitors the internal state of the body (i.e., interoception) (31), but, as mentioned above, it is also a key brain area of the CAN.

Further research linking peripheral ANS responses and the brain are provided by Geisler and Polich (32). These authors found a negative correlation between their participants' heart rates and the P300 latency periods of evoked response potentials (ERPs) generated from auditory cues over parietal cortices. ERPs are visual illustrations of millisecond changes in electrical activity within defined and stimulus-specific brain circuits, and are comprised of several waveform components representing specialized cortical processes (33). The P300 component (a positive deflection peaking approximately 300 ms following stimulus exposure) reflects maintained conscious attentional processing and cortical updating (34, 35). Geisler and Polich also found that their participants' oral temperatures and P300 latency periods in their parietal ERPs were negatively correlated (36). This suggests that the P300 latency period reflects the ANS reactivity of the body, such that the greater the bodily ANS reactivity the shorter the P300 latency period (37).

Given that both Stroop task performance (10) and food cues (6, 7) have been found to modulate ANS reactivity, we thus examined the associations between our participants' P300 latencies (from the ERPs generated by the food/office images) and their cardiac ANS reactivity as measured by heart rates and HRV measures. In addition, since successful weight loss maintainers have been found to have greater brain sensory reactivity to food cues (30), we also investigated group correlations between P300 latencies and heart rates in the weight loss maintainers vs. control participants.

Furthermore, the reward/effort processes underpinning the above cognitive performances/ingestive behavioral prompts has been found to be modulated by both dopamine and noradrenaline neurotransmission (38). Both of these 2 neurotransmitters are distinctly associated with the P200 component of the ERP—a positive deflection peaking approximately 200 ms post stimulus presentation—related to activity which modulates physiological processes underlying the evaluation of stimuli during early attention (39). Given that dopamine and noradrenaline neurotransmission also impacts ANS regulation (40), we investigated the relationship between P200 latencies and HRV measures in our successful weight loss maintainers vs. healthy participants. Liou et al. (41) found correlations between EEG theta power at temporal (T3 and T6) electrodes and HRV measures at rest; and between EEG theta power at the T4 electrode and HRV measures during deep breathing. We thus examined the correlations between the P200 ERP at C3 and C4 electrodes (directly adjacent to the T3 and T4 electrodes) and HRV measures.

In our previous paper we showed that restrained eaters displayed enhanced executive control during *maintained* attentional processing of visual food cues (i.e., during the 300–550 ms period) in an effort to mute the incentive value of external food cues (42). This enhanced executive function displayed by our restrained eaters suggest them to have enhanced anticipatory ANS responses when exposed to visual food cues that need to be suppressed (26).

Our aim was to examine the effects that an attention-demanding Stroop task has on cardiac ANS reactivity to visual food cues in healthy women. The women were split into two groups, made up of individuals who maintained clinically relevant weight loss (CWL; ≥5% of body mass) for at least 12 months and BMI matched normal weight individuals who had never weight cycled (CTL). Given that successful weight loss maintainers have (1) alterations in brain processing of food cues (30) and (2) engage in different eating behavior strategies (25) vs. individuals of normal weight and (3) that these differences are influenced by stress (29), we examined whether visual food cues had a different effect on cardiac ANS reactivity associated with performing a cognitive task in successful weight loss maintainers vs. control participants.

Our hypotheses were set out as follows:

**The** ***presence of calorie dense food images*** vs. neutral images during a Stroop task would have significant modulating effects on:

(1a) a*ttention processing speeds*, specifically that there will be a relationship between P300 ERP latency periods and heart rates and/or HRV measures and (1b) that the P300 ERP latency vs. heart rate modulation will be different in CWL vs. CTL participants;
(2a) e*arly attention processing speeds*, specifically that there will be a relationship between P200 ERP latency periods and heart rate and/or HRV measures and 2b) that the P200 ERP latency vs. heart rate modulation will be different in CWL vs. CTL participants;
(3a) participants' *heart rate and HRV* 3b) and it would have a significantly greater modulating effect in CWL vs. CTL participants;

## Materials and Methods

### Research Participants

A convenience sample of apparently healthy^*^ women was recruited via local primary school newsletters, through social media, and notice board announcements at a commercial wellness center. Participants were excluded if they reported a history of a known metabolic disease, pregnancy or lactation in the last 3 months. The women were allocated to a clinical weight loss (CWL) group, or to a body mass index (BMI)-matched control (CTL) group with no reported history of clinically meaningful weight reduction. The experimental protocol was approved by the University of Cape Town Faculty of Health Sciences Human Research Ethics Committee (HREC reference: 214/2012), and all participants provided informed consent prior to assessment. Participants were tested at our EEG laboratory situated at Valkenberg Hospital, Observatory, Cape Town.

^*^Here the term “apparently healthy” refers to an absence of diagnosed chronic disease (mental or physical) which may confound our results. These individuals were identified via a standardized inclusion/exclusion screening questionnaire.

### Anthropometry, Demographics, and Retrospective Data

Weight (BW-150, NAGATA, Tainan, Taiwan), height (3PHTROD-WM, Detecto, Missouri, USA), waist circumference, and hip circumference measurements were collected. A demographic questionnaire verified socio-cultural and economic similarity between groups, and a health questionnaire was implemented to exclude applicants with known metabolic disease, eating pathology, positive HIV status, and chronic medication usage. A reproductive survey was administered to exclude women presenting with menstrual dysfunction, hysterectomy or menopause.

### Eating Behavior Questionnaire

The validated 51-item Three Factor Eating Questionnaire (TFEQ) was employed to measure dietary restraint, disinhibition, and trait-related hunger. Higher scores being indicative of greater degrees of dietary restraint, disinhibition and hunger. The reason we chose the TFEQ was to compare our data to existing data (the TFEQ has been used widely in the South African literature pertaining to obesity) and also for us to draw parallels with work published on the National Weight Control Registry (NWCR) in the U.S. Furthermore the TFEQ provides a means to ensure homogeneity between our participants and to allow for correlation-based analyses.

### Electrocardiographic Recordings

ECG activity was recorded from 3 electrodes (Blue Sensor, Ambu, Denmark) placed in positions representing Eindhoven's triangle namely, subclavicular bilaterally and over the left anterior superior iliac crest. The skin surface was cleaned and gently abraded with an alcohol swab before electrodes were attached. Electrode cables were taped down to prevent movement artifact. The 3 electrodes were connected to a Biopac MP150 system (Goleta, CA 93117, USA) ECG amplifier set to band-pass filter between 0.5 and 35 Hz and a sampling frequency of 500Hz. ECG recordings were analyzed with AcqKnowledge for Windows (version 4.1). The filtered ECG recording tachograms were then visually inspected to determine the correct recognition of QRS complexes and T waves. Missed and ectopic beats were corrected by either adding or spacing beats (43).

### Heart Rate Variability (HRV) Analyses

Only after each tachogram showed no spurious beats were the data analyzed using HRV analysis software (Kubios v 2.1) from the Biomedical Signal Analysis Group (Department of Applied Physics, University of Kuopio, Finland). Data were transformed using autoregressive (AR) analysis, with an AR model order of 15, into low frequency (LF) (0.04–0.15 Hz) and high frequency (HF) (0.15–0.4 Hz) components (44). We completed our statistical analyses on both frequency [LF power (LF power), HF power (HF power)] and the time domain measure, root mean square of successive differences (RMSSD). The frequency domain power values in ms2 were log transformed to normalize this data as log LF power and log HF power. Total power in the heart (log TF) was taken as log (LF power + HF power).

### Breathing Rates

The breathing rate per minute was measured via a Biopac force transducer fixed to a belt placed around the chest wall. Subjects were asked to expel the air from their lungs when the transducer belt was first fitted and then instructed to breathe normally. The chest transducer was connected to a Biopac MP150 RSP100C amplifier with a low-pass 10 Hz filter. Breathing rates were manually counted.

### Electroencephalographic (EEG) Event-Related Potentials (ERPs)

EEG signals were sourced from 10 scalp sites (Fp_1_, Fp_2_, F_3_, F_4_, F_7_, F_8_, C_3_, C_4_, P_3_, P_4_) with EEG cap (http://electro-cap.com/) arranged via the international 10/20 montage system using 10 EEG Biopac amplifiers connected to the MP150 system and ear-linked reference electrodes (A_1_, A_2_). Preceding extraction, the raw EEG data were corrected for EOG artifact (automated ICA) within Acqknowledge 4.1. ERP waveforms were extracted using an automated Matlab-designed program (Matlab, Mathworks, MA, USA). The data were band pass filtered (FIR) with a Hamming window of 0.1–30 Hz. ERP Epochs were set at 200 ms prior to and 600 ms post cue presentation to capture an 800 ms window. Extraction was set to reject ERPs ± 100 μV where each subject must have presented viable ERPs within rejection limits for ≥75% of image trials (i.e., no less than 15 out of 20) of food and office image trials to be included in the analyses which followed. ERPs were baseline corrected, and grand average waveforms were generated to identify robust ERP components. Latency (ms) values were obtained for each ERP wave component. A detailed description of the components extracted (and their respective windows) are outlined in an earlier work (45). Of relevance to the current report are the P200 and P300 windows of extraction. Components extracted around image exposure included P200-like (200–300 ms window, central electrodes (C_3_, C_4_)) and P300-like (200–550 ms window in parietal electrodes (P_3_, P_4_)) components. Geisler and Polich (32, 36) found correlations between the P300 ERP recorded at the midline parietal electrode (Pz) and heart rates and oral temperatures, while Liou et al (41) found correlations between EEG theta power at temporal electrodes at rest and during deep breathing. We did not measure ERPs at temporal electrodes, instead using data recorded at the C_3_ and C_4_ electrodes directly adjacent to the T_3_ and T_4_ electrodes.

### Modified Stroop Tasks

We modified the original Stroop task by programming ePrime software (PST, Sharpsburg, PA, USA) to display the color word conflicts and embedded images between the color words. As described previously (43) three Stroop tasks were conducted with record of EEG, ECG and breathing rate, where each task included 20 embedded images of one nature (white squares or high calorie food or office stationary/furniture (non-food-related, i.e., neutral images) items). The first Stroop task that participants completed was a full familiarization Stroop task embedded with 20 white squares. Participants were asked to count how many white squares appeared during the task as they would have to recall this (verbally) at the end of each task, thereby including loading of working memory during these tasks. Immediately after completing the practice Stroop task the participants randomly completed a Food Stroop task (containing 20 embedded food pictures) and a neutral Office Stroop task (containing 20 embedded office pictures). Words and images appeared every 3 s for 400 ms and were then replaced by a blank screen which was the response period that lasted 2600 ms. A total of 95 cues were presented randomly per task: sixty incongruent color words (15 of each color, red, blue, yellow and green), 15 gray words and 20 white squares or food/office pictures, depending on which modified Stroop task was being completed. All images were comparable for quality and brightness, with food images included only if sold at local supermarkets. Food and neutral tasks were included to deduce whether differences in behavioral or electrophysiological data were attributable to the nature of a cue (i.e., food vs. office) and were not merely as a result of exposure to any inclusive image. Mean reaction time was calculated for correct responses. To control for outliers, mean reaction times for each group (i.e., CWL and CTL) and for each task (food and non-food) were calculated with the exclusion of participants yielding values ≤ 200 ms or ≥2000 ms, or reaction times exceeding the population mean by ± 2 standard deviations (SD).

### Procedure

The experimental design has been published (45) that reported ERP differences in normal weight, overweight and obese individuals. Thus, only a brief overview of the methodology is provided here that address the additional components investigated in this report. Upon arrival anthropometric data were collected, physiological recording devices attached where-after participants subjectively indicated their momentary satiety and prospective hunger levels via a visual analog scales (VAS). Participants were then asked to execute three modified Stroop tasks with record of electroencephalography (EEG), electrocardiography (ECG) and breathing rates: a familiarization task that included 20 white squares, that had to be mentally counted, amongst the color words, followed by a food- or office-related task at random. Data were captured between 12h00 and 16h00, and all participants were naïve to the testing session.

### Statistical Analysis

Stata 12 software package (Stata, StataCorp, TX, USA) was used for statistical analyses. Shapiro-Wilk W tests were conducted to examine data normality. Student's *t*-tests were performed to assess for differences in parametric data and Pearson pairwise correlations were calculated between the parametric HRV measures and ERP latencies. Mann-Whitney U tests were performed to examine non-parametric data. The assumptions of statistical tests regarding the homogeneity of variances were tested with Bartlett's test for parametric data and Levene's test for non-parametric data. Parametric data in Table and text are described as means ± standard deviation (SD). Alpha was set at <0.05.

## Results

### Physical Characteristics

Forty five women with no known illness or disorder, similar socioeconomic status, BMI and age participated in the study. Five of the 45 women were excluded from subsequent data analyses, 3 due to BMIs > 2 SDs more the mean, 1 due to being hypertensive (148/84 mmHG and >2 SDs higher than mean) and the Stroop reaction times of 1 participant was >2 SDs slower than the mean. Of the remaining 40 women 17 retained a clinically meaningful weight loss (≥5% of body weight) for a period of at least 12 months by non-surgical means, while 23 reported no history of clinically relevant weight reduction. No differences were found between groups for BMI, weight (kg), height (cm), BP or hip circumference (cm) (Table 1).

**Table 1:** Subject characteristics, Three Factor Eating Questionnaire (TFEQ) scores and pre-trial VAS scores: CWL vs. CTL groups.

	**All**	**CWL (*n* = 17)**	**CTL (*n* = 23)**
Age (years)	30.9 ± 7.4	30.9 ± 6.4	30.8 ± 8.2
Weight (kg)	165.4 ± 7.1	166.3 ± 8.6	164.7 ± 5.8
Height (cm)	70.1 ± 12.8	66.4 ± 9.6	72.8 ± 14.3
BMI (kg/m2)	25.7 ± 4.8	24.0 ± 3.1	26.9 ± 5.5
Waist/hip ratio	0.71 ± 0.05	0.69 ± 0.05	0.72 ± 0.06
Systolic BP	114 ± 10	111 ± 8	116 ± 11
Diastolic BP	72 ± 9	68 ± 7	75 ± 9
TFEQ restraint factor	8.6 ± 4.1	9.9 ± 4.0	7.6 ± 4.1
TFEQ disinhibition factor	7.8 ± 4.2	6.8 ± 4.1	8.4 ± 4.3
TFEQ hunger factor	5.9 ± 3.8	5.7 ± 3.8	6.0 ± 3.8
Hunger (VAS)	43 ± 27	47 ± 25	40 ± 28
Fullness (VAS)	46 ± 29	42 ± 29	48 ± 29
Desire to eat (VAS)	46 ± 26	56 ± 23	39 ± 26^*^
Quantity (VAS)	36 ± 19	34 ± 18	37 ± 20
Satiety (VAS)	52 ± 27	46 ± 31	57 ± 24

Desire to Eat significantly higher in CWL vs. CTL,

* *p < 0.05*.

### Self-Report Questionnaires

#### Eating Behaviors

**Figure 1:**
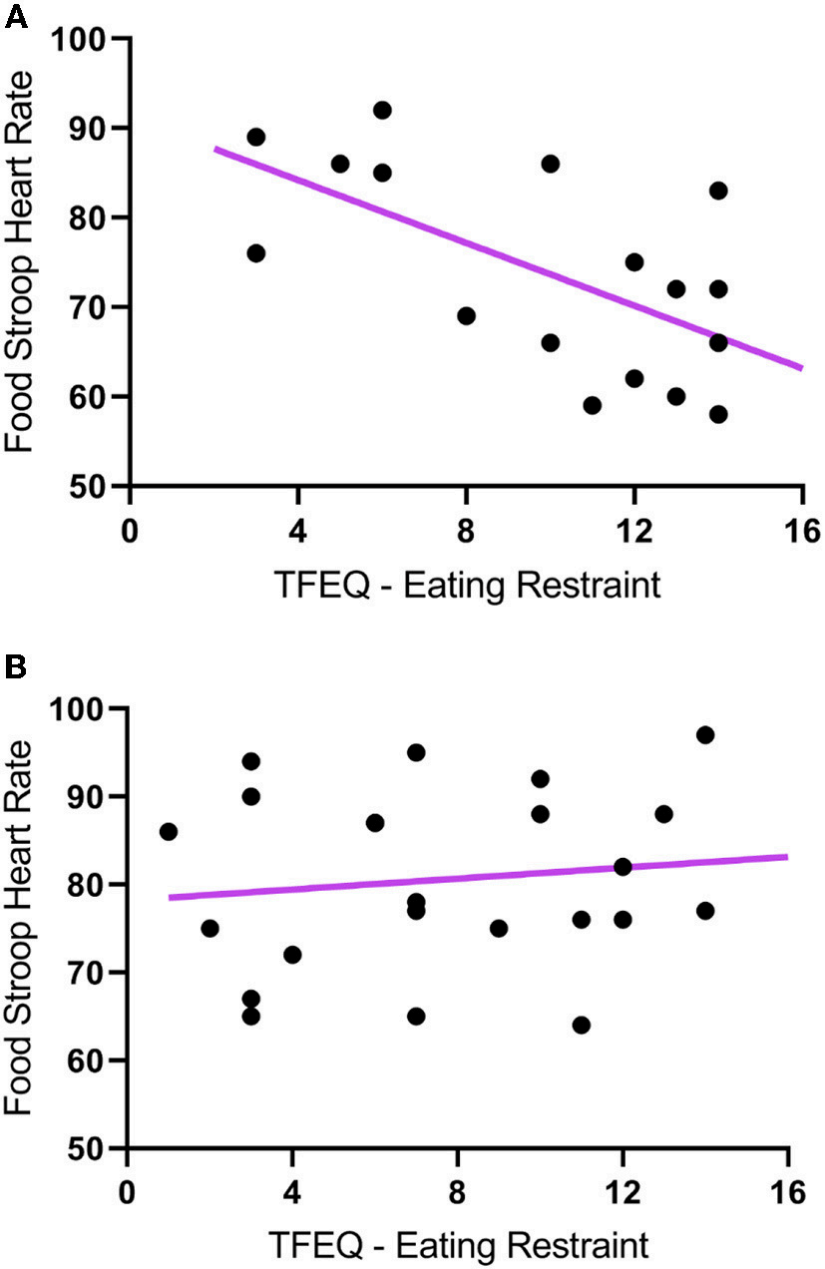
The correlations between Eating Restraint scores (Three Factor Eating Questionnaire) and heart rates recorded during the Food Stroop tasks. **(A)** clinically relevant weight loss (CWL; ≥5% of body mass) group (*r* = 0.62, *p* < 0.01) and **(B)** women who had never weight cycled (CTL).

The TFEQ yielded no differences between groups for dietary disinhibition or perceived hunger or eating restraint Table 1. However, we did find a significant negative correlation between CWL participants' eating restraint scores and their heart rates during the Food Stroop (*r* = 0.62, *p* < 0.01, Figure 1A) and the Office Stroop tasks (*r* = 0.60, *p* < 0.02; data not shown). These relationships were not found in the CTL group (Figure 1B).

#### Pre-experimental Satiety

Only one of the subjective pre-experimental hunger/satiety ratings was different between groups, momentary Desire to Eat (VAS) was significantly higher in CWL participants prior to the performance of Stroop Tasks with record of EEG, ECG and breathing rates (p < 0.05, Table 1). This was despite participants having had refrained from food and beverage intake for similar periods of time.

### Stroop Task Reaction Time and Accuracy Scores

No differences in behavioral measures of Stroop task performance (i.e., reaction time, number of incorrect color-word responses, and picture counting) were found (Table 2).

**Table 2:** Office and Food Stroop task reaction times, mistakes made and images counted: CWL vs. CTL groups.

	**All**		**CWL (*****n*** **=** **17)**		**CTL (*****n*** **=** **23)**	
	**Office**	**Food**	**Office**	**Food**	**Office**	**Food**
Reaction time (ms)	698 ± 167	685 ± 159	735 ± 150	728 ± 166	674 ± 177	656 ± 152
Mistakes	2 ± 2	2 ± 2	2 ± 2	2 ± 1	2 ± 2	2 ± 2
Pictures counted	20 ± 2	20 ± 2	20 ± 2	20 ± 2	20 ± 1	19 ± 2

### ERP Wave Components (P200 and P300) Correlations With Cardiac ANS Reactivity During the Food vs. Office Stroop Tasks

**Figure 2:**
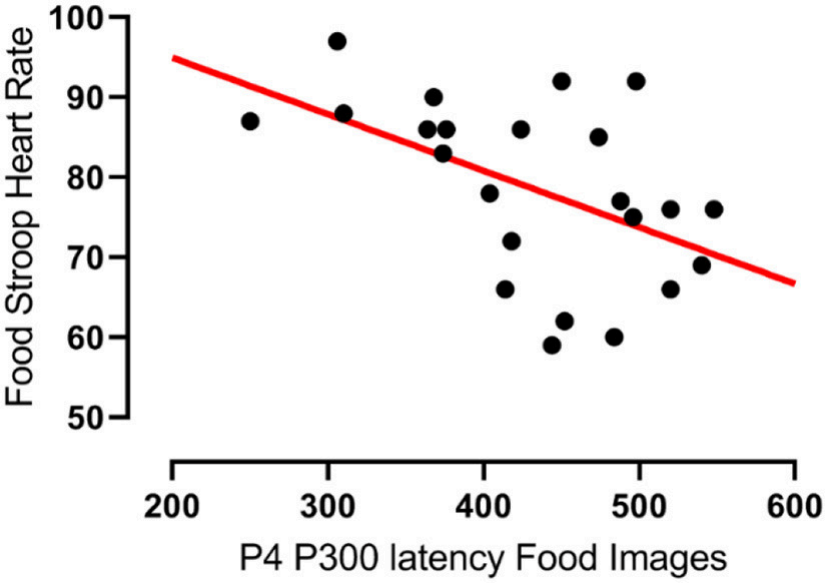
Correlation between the latency of participants' P300 components evoked by food cues at the right parietal (P_4_) electrode and their heart rates during the Food Stroop task. r = −0.51, *p* < 0.02.

In agreement with hypothesis 1a there was a significant negative correlation between the latencies of participants' P300 component evoked at the right parietal (P_4_) electrode upon food image presentation and their heart rates during the Food Stroop Task (*r* = −0.51, *p* < 0.02, Figure 2). Contrary to hypothesis 1b there were no group differences.

**Figure 3:**
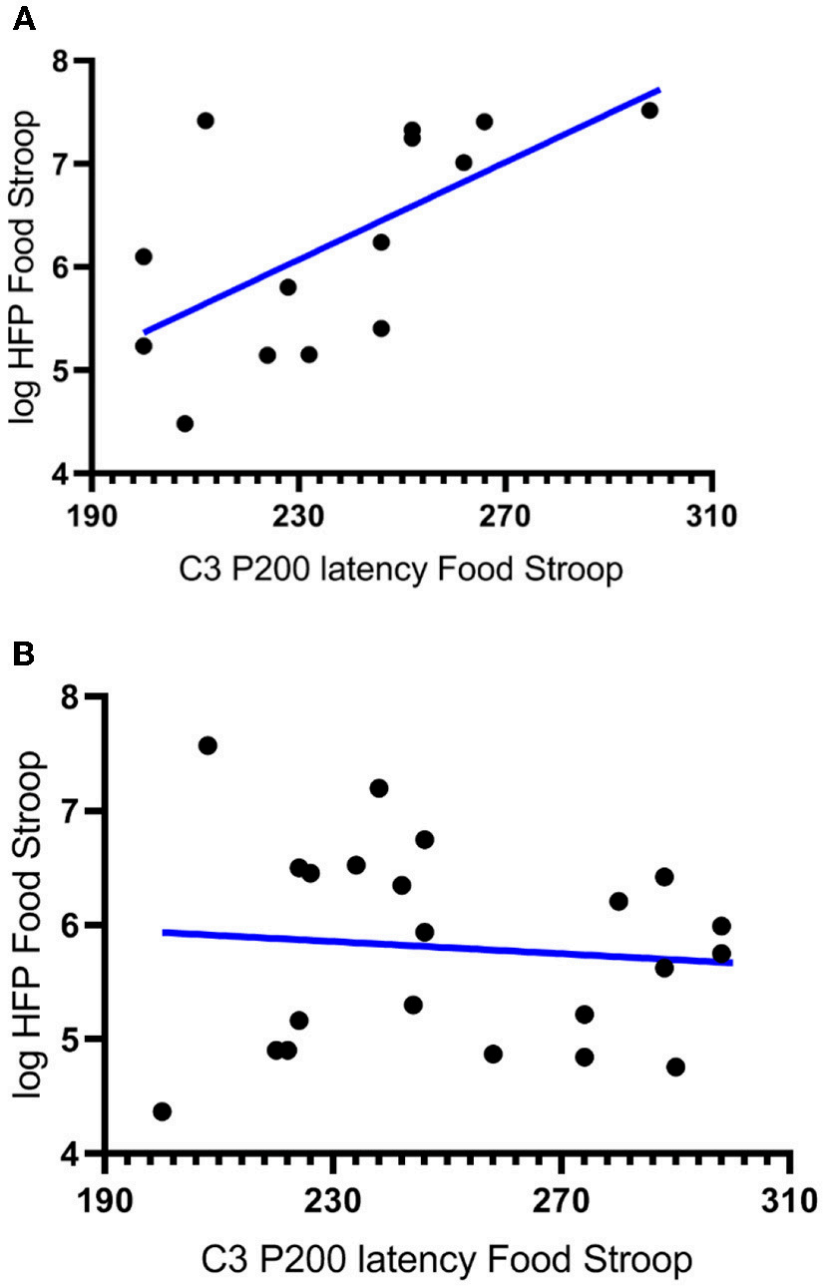
Correlations between the latency of participants' P200 components evoked by Stroop cues at the left central (C_3_) electrode and the log High Frequency power (logHFP) in their cardiac spectrograms during the Food Stroop. **(A)** clinically relevant weight loss (CWL; ≥5% of body mass r = 0.63, *p* < 0.02) group and **(B)** women who had never weight cycled (CTL).

In partial agreement with hypothesis 2*a* there was a significant negative correlation between the latencies of the CWL participants' P200 component evoked at the left central (C_3_) electrode upon Stroop image presentation and the logHFpower in their cardiac spectrograms during the Food Stroop Task (*r* = 0.63, *p* < 0.02, Figure 3A). In agreement with hypothesis 2*b* there was no such correlation in the CTL group.

### Heart Rate, HRV Measures and Breathing Rates During the Food Stroop vs. Office Stroop

*Task differences:* In agreement with hypothesis 3a, most of the cardiac ANS variables measured, heart rate, RMSSD and log Total power (logTP) were significantly different during the Food vs. Office Stroop Tasks (Table 3). Breathing rates fell into the high frequency band of the cardiac spectrogram and they were similar during both the Office and Food Stroop tasks showing that the spontaneous breathing rates did not have different impacts on frequency domain HRV measures.

**Table 3:** Cardiac ANS reactivity: Food vs. Office Stroop tasks when groups were combined.

	**Office stroop**	**Food stroop**
Resting heart rate (bpm)	66 ± 9^#^	6.6 ± 9^#^
Stroop heart rate (bpm)	79 ± 11	78 ± 11^*^
Breathing rate (breaths/min)	19 ± 3	19 ± 3
Stroop rmssd (ms)	32 ± 16	34 ± 16^**^
Log high frequency (hf) power (ms^2^)	5.8 ± 10	5.9 ± 0.9
Log low frequency (lf) power (ms^2^)	5.7 ± 0.9	5.8 ± 0.9
Log total (hf + lf) power	6.5 ± 0.8	6.7 ± 0.8^**^

*Food Stroop significantly different from Office Stroop*,

* p < 0.05,

** *p < 0.01 Bonferroni corrected*.

*Resting heart rate different from Stroop task heart rates*,

# *p < 0.000*.

*Group differences*: Contrary to hypothesis 3b there were no between group differences for any of the cardiac ANS measures during the two Stroop Tasks (Table 4).

**Table 4:** Cardiac ANS reactivity in clinical relevant weight loss (CWL; ≥5% of body mass) participants compared to women who had never weight cycled (CTL) during Food vs. Office Stroop tasks.

	**CWL (*****n*** **=** **19)**		**CTL (*****n*** **=** **23)**	
	**Office**	**Food**	**Office**	**Food**
Stroop heart rate (HR)	76 ± 12	74 ± 11	81 ± 9	81 ± 10
Stroop RMSSD (ms^2^)	37 ± 19	39 ± 20	29 ± 12	30 ± 13
log total (HF + LF) power	6.7 ± 0.9	6.9 ± 0.8	6.4 ± 0.8	6.5 ± 0.8

## Discussion

Our first finding was that despite there being no group differences in TFEQ eating restraint, we nevertheless found a negative correlation between eating restraint and the CWL participants' heart rates during the Stroop tasks (Figure 1A, this was not apparent in CTL–Figure 1B). Second, we found a correlation in our CWL participants during the high-calorie food image Stroop; their P200 latencies evoked by the Stroop cues recorded at the C3 electrode was positively correlated to log High Frequency power in their cardiac spectrograms (Figure 3A this was not apparent in CTL–Figure 3B, and not apparent in either group for neutral image Stroop). Our third finding was for all participants, where there was a negative correlation between the P4 electrode P300 latencies evoked by the high-calorie food images and their heart rates, but there were no group differences in this correlation (Figure 2).

Our last finding was that cardiac ANS responses in our participants were dampened during the high-calorie food image Stroop task relative to the neutral image Stroop task (Table 3), but we found no group differences (Table 4). This was evidenced by lower heart rate and higher RMSSD and log Total power in the participants' cardiac spectrograms when they completed the Stroop task embedded with high calorie food prompts vs. the Stroop task embedded with neutral office furniture prompts. Further to this point, the significant increases in heart rate during both Stroop tasks vs. at rest (Table 3) supports that the Stroop task(s) were associated with significant dampening of cardiac vagal drive (46), and presumably sympathetic nerve activation (47), relative to resting ANS activations.

This provides evidence that the presence of 20 visual food cues during the completion of a cognitive performance test (that was associated with significantly increased heart rates, *p* < 0.001), had a significant dampening effect on the attendant cardiac ANS reactivity (43, 48). More specifically the significantly lower heart rates and higher HRV during the Food vs. Office Stroop tasks are indicative of enhanced vagal drive (49, 50) during the Food Stroop. Indeed, food cue exposure result in so-called “cephalic phase responses” that primes the gut for digestion and absorption (21). These cephalic phase responses are mediated via rostral forebrain modulation of the dorsal vagal complex (DVC) in the brainstem (21, 50). The DVC then integrates the information from the rostral forebrain with inputs from visceral afferents to control digestive and ingestive processes via parasympathetic motor fibers (51).

This is opposite to the ANS responses evoked by food cues on their own, i.e., when not embedded within a cognitive performance test (6, 7). Food cue exposure in participants at rest was associated with increased heart rate, blood pressure and electro-dermal skin response (6, 7). The subsequent food intake in these participants correlated with their subjective craving, which in turn correlated with their diastolic and systolic blood pressure changes (6). Our apparently contradictory findings can be put down to the activation of anticipatory ANS responses upon food cue exposure (4) reported in Nederkoorn and Jansen 2004 (6) and Vögele and Florin 1997 (7) studies. This is evidenced by the increased hand grip force observed in participants wanting to obtain high vs. low calorie food items (52). This increase in ANS reactivity due to food cues relates to the bodily preparations needed for foraging behavior to obtain food (10).

While it is well known that stress and eating are linked (16–18, 53, 54), less is known about the physiological underpinnings of this link. Our study is the first to demonstrate that the mere presence of food cues during a cognitive performance test (associated with significant heart rate increase), resulted in decreased heart rate and increased HRV. This provides another neurobiological driver for stress induced eating (17). Interestingly we found no difference in the ANS reactivity to food cues in CWL vs. CTL participants, even though the momentary desire to eat was significantly higher in the CWL vs. CTL participants (56 ± 23 vs. 39 ± 26, *p* < 0.02, Table 1).

This ties in with the significant positive correlation we found between the P200 latencies at the C_3_ electrode upon Stroop cue exposure in CWL participants and the log HF power in their cardiac spectrograms during the Food Stroop task (Figure 3). Presumably the early attention processing (P200) correlation we found over the left hemisphere in CWL participants may be related to parasympathetic nervous system (PSNS) modulation of the heart (55). Indeed, Hilz et al. (55) found that the PSNS predominates in the left hemisphere of their test populsation (epilepsy patients), which ties in with our finding of greater log HF power in the cardiac spectrogram (which is a good marker of cardiac PSNS activation) of those CWL participants who had slower P200 latencies upon Stroop cue exposure. This close link between the brain and the heart during early attention processing in the CWL participants during a cognitive performance task may well account for the enhanced executive control we previously found in restrained eaters during attentional processing of visual food cues (42). It may also explain why restrained eaters succumb when allocating cognitive resources to an attention-demanding task that overstretch their ability to monitor dietary restraint as proposed by Wallis and Hetherington (29). Finally it may also explain why those CWL participants who had lower heart rates during Stroop task performance had higher eating restraints scores (Figure 1A). Presumably similar neural processes account for the enhanced executive control over bodily prompts at rest (Eating Restraint) than during Stroop performance (greater cardiac vagal drive).

Our third finding, a negative correlation between the latencies of participants' P300 wave at the right parietal (P_4_) electrode evoked by food cue exposure and their heart rates, agrees with Geisler & Polich's findings (32) in that shorter P300 latensies (faster attentional processing) of food cues are associated with higher heart rates (Figure 2). Both groups had similar, yet non-significant, correlations that only became significant when the groups were combined. There was only a trend (*r* = 0.29, *p* = 0.083) for such a correlations in the combined two groups during the Office Stroop task, which suggests that the food cues had an additional modulating effect on attention processing in all participants completing the Food Stroop task.

The following limitations may have impacted the results. The images were sourced from the internet, rather than from a validated database and we did not pilot test the images. Instead the researchers compared the images for quality and brightness. This was an oversight on our part that has since been corrected. Given that our selected food cues evoked several significant EEG and heart rate variability modulatory effects in our participant groups, we are confident that they very of high enough quality and brightness for our research purposes. A further limitation was that we did not directly measure how stressed the participants felt regarding completing the Stroop tasks. Therefore we had to infer, from the very significant heart rate increases during the Stroop tasks vs. at rest and from previous Stroop task studies, that the participants would have felt stressed during the Stroop task.

In conclusion there was a significant correlation between P300 latencies evoked by food cues and heart rate across all participants. There were also 2 significant correlations in CWL participants that were not found in the CTL participants, (1) between baseline Eating Restraint scores and heart rates during the Stroop tasks and (2) between P200 latencies evoked by Stroop cues and the high frequency power in the participants' cardiac spectrograms during the Food Stroop task. This provides evidence that even 12 months after successful weight loss maintenance the cardiac ANS reactivity to food cues during a cognitive performance test was still altered in CTL participants. Finally, across all participants the cardiac ANS reactivity evoked by completing the Stroop color-word conflict task was lowered by food cues suggesting that this dampening effect that food cues has on cardiac ANS reactivity may form part of the physiological pathway underpinning “stress induced” eating.

## Author Contributions

HR co-developed research, wrote part of protocol, was responsible for HRV analysis/design and wrote the first draft. DH co-developed, co-wrote protocol, executed research, and was responsible for data collection/analysis/design, EEG write up and editorial input. FH was responsible for EEG analysis/design and EEG write up. JK co-developed research, and contributed editorial input. EL conceptualized the idea for the research, co-wrote protocol and provided research funding and editorial input. All authors have approved the final manuscript.

### Conflict of Interest Statement

The authors declare that the research was conducted in the absence of any commercial or financial relationships that could be construed as a potential conflict of interest.
